# Structural and Bioactivity Characterization of Filipin Derivatives from Engineered *Streptomyces filipinensis* Strains Reveals Clues for Reduced Haemolytic Action

**DOI:** 10.3390/antibiotics9070413

**Published:** 2020-07-16

**Authors:** Eva G. Barreales, Ángel Rumbero, Tamara D. Payero, Antonio de Pedro, Ester Jambrina, Jesús F. Aparicio

**Affiliations:** 1 Department of Molecular Biology, Area of Microbiology, Faculty of Biology, Universidad de León, 24071 León, Spain; egarcb@unileon.es (E.G.B.); tdiep@unileon.es (T.D.P.); apedl@unileon.es (A.d.P.); ejambri92@gmail.com (E.J.); 2 Department of Organic Chemistry, Faculty of Sciences, Campus de Cantoblanco, Universidad Autónoma de Madrid, 28049 Madrid, Spain; angel.rumbero@uam.es

**Keywords:** antifungal agent, polyene macrolide, *Streptomyces filipinensis*

## Abstract

The rise in the number of immunocompromised patients has led to an increased incidence of fungal infections, with high rates of morbidity and mortality. Furthermore, misuse of antifungals has boosted the number of resistant strains to these agents; thus, there is urgent need for new drugs against these infections. Here, the in vitro antifungal activity of filipin III metabolic intermediates has been characterized against a battery of opportunistic pathogenic fungi—*Candida albicans*, *Candida glabrata*, *Candida krusei*, *Cryptococcus neoformans*, *Trichosporon cutaneum*, *Trichosporon asahii*, *Aspergillus nidulans*, *Aspergillus niger*, and *Aspergillus fumigatus*—using the Clinical and Laboratory Standards Institute broth microdilution method. Structural characterization of these compounds was undertaken by mass spectrometry (MS) and nuclear magnetic resonance (NMR) following HPLC purification. Complete NMR assignments were obtained for the first time for filipins I and II. In vitro haemolytic assays revealed that the haemolytic action of these compounds relies largely on the presence of a hydroxyl function at C26, since derivatives lacking such moiety show remarkably reduced activity. Two of these derivatives, 1′-hydroxyfilipin I and filipin I, show decreased toxicity towards cholesterol-containing membranes while retaining potent antifungal activity, and could constitute excellent leads for the development of efficient pharmaceuticals, particularly against *Cryptococcosis*.

## 1. Introduction

In the last few decades, there has been a continuous rise in the incidence of fungal infections with deadly results [1]. This is largely due to the increase in the population of immunocompromised individuals. Several factors contribute to such increase, such as HIV infection, tuberculosis, leukaemia, immunosuppressive therapy, cancer chemotherapy, organ transplantation, or the use of broad-spectrum antibiotics [2]. In these individuals, a common infection can easily become fatal if the disease is prolonged [3]. In addition, modern medical interventions have extended the survival of patients in critical condition, who are also highly vulnerable to this type of infections [4]. Despite the unacceptably high mortality rates of invasive fungal infections, these illnesses are not investigated enough compared to other microbial infections [3]. Furthermore, there is also an increasing frequency of resistant fungal strains to the available treatments [5]. Thus, there is an urgent need for novel antifungal drugs.

Among the different diseases caused by fungi, candidiasis is the most worrying, due to its worldwide incidence [6]. Together with given *Candida* species (mainly *C. albicans*, *C. krusei*, and *C. glabrata*), *Cryptococcus neoformans*, and members of the genus *Aspergillus* (responsible for meningeal cryptococcosis and invasive aspergillosis, respectively), as well as *Histoplasma capsulatum* and *Pneumocystis jirovecii*, are the cause of about 90% of deaths related to fungal infections, killing more than 1.6 million people every year [1]. Although they have lower incidence, infections caused by *Fusarium* and *Trichosporon* have also become important, with potentially lethal outcomes in immunocompromised patients [7,8].

**Figure 1:**
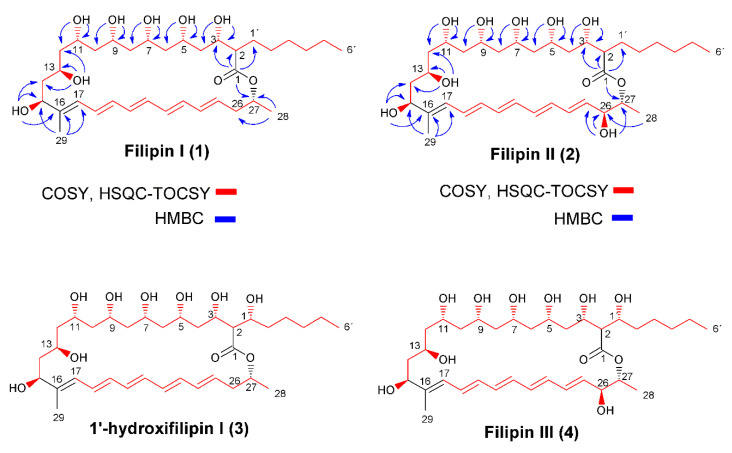
Chemical structures of filipin III and its precursors. COSY (H,H-correlation spectroscopy) and heteronuclear single quantum coherence (HSQC)-TOCSY correlations, as well as key heteronuclear multiple-bond correlation (HMBC) correlations for filipin I (1) and filipin II (2) are included.

Filipin III (4) is a potent antifungal polyene macrolide, widely used for the detection and the quantitation of cholesterol in biological membranes [9], and as a tool for the diagnosis of Niemann–Pick type C disease [10]. Unlike most polyene macrolides, which display significantly higher affinity for ergosterol than for other sterols, filipin III also has a high affinity for cholesterol [11], and therefore interacts with the cell membranes of mammals. This precludes its use in medical therapies with humans, due to its derived toxicity. We previously studied its biosynthesis in the industrial producing strain *Streptomyces filipinensis*, and by genetic manipulation of two cytochrome P450 monooxygenase encoding genes (*filC* and *filD*, responsible for hydroxylations at positions C26 and C1′ respectively), we revealed the existence of two alternative routes for its formation [12]. Using the generated mutant strains, we managed to produce filipin III derivatives as shunt metabolites (Figure 1). These derivatives differ in the substituents of the C1′ and C26 carbons of the molecule. While filipin I (1) lacks substituents in both positions, 1′-hydroxyfilipin I (26-deoxyfilipin III) (3) and filipin II (1′-deoxyfilipin III) (2) have a hydroxyl group at the positions C1′ or C26, respectively (Figure 1). Among all these derivatives, 1′-hydroxyfilipin I is the only one that is not produced in nature by *S. filipinensis* [12]. Preliminary studies have shown that all these intermediates display antifungal activity against *C. utilis* and *Saccharomyces cerevisiae*, and some of them are enhanced when compared with filipin III [12]. Here, we extend these preliminary studies on some opportunistic pathogenic fungi of medical interest, and analyse the haemolytic capacity that these compounds possess on mammal cells, finding that some of them show decreased toxicity towards cholesterol-containing membranes while retaining potent antifungal activity. Thus, these compounds could constitute pharmaceuticals with improved biological properties. Complete nuclear magnetic resonance (NMR) assignments have been obtained for the first time for filipin I and filipin II.

## 2. Results

### 2.1. Structural Characterization of Filipin III Precursors

Filipin I was obtained from fermentation broths of *S. filipinensis ΔfilCD*, whereas we used *S. filipinensis ΔfilD* and *S. filipinensis ΔfilC* for the purification of filipin II and 1′-hydroxyfilipin I, respectively [12]. Polyenes were purified as described in Materials and Methods. Structures of filipin III precursors were then established by mass spectrometry (MS), followed by one-dimensional (1D) and two-dimensional (2D) NMR spectroscopy. The structure of 1′-hydroxyfilipin has already been described [12], but to our knowledge this is the first report of the complete NMR assignments for filipin I and filipin II (26-hydroxyfilipin I).

Filipin I’s molecular formula was established as C_35_H_58_O_9_ by MS (*m/z* 645.5 [M+Na]^+^), ^13^C NMR, and ^1^H NMR (Figure S1). Analysis of ^13^C NMR, distortionless enhancement by polarization transfer (DEPT), and heteronuclear single quantum coherence (HSQC)/heteronuclear multiple quantum correlation (HMQC) data (Figure S1) showed the presence of 24 sp^3^ carbon signals (three CH_3_, 12 CH_2_, and nine CH) and 11 sp^2^ carbon signals. The NMR data are summarized in Table 1.

The ^1^H NMR, DEPT, and H,H-correlation spectroscopy (COSY) spectra (Figure S1) revealed seven oxygenated methine protons at δ_H_ 3.61 (m, 1H, H-3) and δ_OH_ 5.05 (d, *J* = 4.8 Hz, OH-3), δ_H_ 3.83 (m, 1H, H-5) and δ_OH_ 4.97 (d, *J* = 2.2 Hz, OH-5), δ_H_ 3.85 (m, 1H, H-7) and δ_OH_ 4.89 (d, *J* = 1.8 Hz, OH-7), δ_H_ 3.82 (m, 1H, H-9) and δ_OH_ 5.13 (s br, OH-9), δ_H_ 3.79 (m, 1H, H-11) and δ_OH_ 4.84 (d, *J* = 2.2 Hz, OH-11), δ_H_ 3.18 (m, 1H, H-13) and δ_OH_ 4.46 (d, *J* = 4.8 Hz, OH-13), and δ_H_ 3.98 (m, 1H, H-15) and δ_OH_ 4.82 (d, *J* = 3.7 Hz, OH-15); nine methine protons of conjugated double bonds at δ_H_ 5.93 (d, *J* = 11.2 Hz, 1H, H-17), δ_H_ 6.46 (dd, *J* = 14.5 and 11.2 Hz, 1H, H-18), δ_H_ 6.23 (m, 1H, H-19), δ_H_ 6.35 (m, 1H, H-20), δ_H_ 6.31 (m, 1H, H-21), δ_H_ 6.18 (m, 1H, H-22), δ_H_ 6.28 (m, 1H, H-23), δ_H_ 6.13 (dd, *J* = 14.5 and 11.2 Hz, 1H, H-24), and δ_H_ 5.75 (dt, *J* = 14.5 and 6.9 Hz, 1H, H-25); two methine protons at δ_H_ 2.21 (m, 1H, H-2) and δ_H_ 5.02 (m, 1H, H-27); 12 methylene protons at δ_H_ 1.30 (m, 2H, H-4), δ_H_ 1.29 (m, 2H, H-6), δ_H_ 1.38 and 1.28 (m, 2H, Ha-8 and Hb-8), δ_H_ 1.35 and 1.22 (m, 2H, Ha-10 and Hb-10), δ_H_ 1.59 and 1.28 (m, 2H, Ha-12 and Hb-12), δ_H_ 1.71 and 1.52 (m, 2H, Ha-14 and Hb-14), δ_H_ 2.35 and 2.25 (m, 2H, Ha-26 and Hb-26), δ_H_ 1.68 and 1.44 (m, 2H, Ha-1′and Hb-1′), δ_H_ 1.24 (m, 2H, H-2′), δ_H_ 1.19 (m, 2H, H-3′), δ_H_ 1.22 (m, 2H, H-4′), and δ_H_ 1.24 (m, 2H, H-5′); three methyl groups at δ_H_ 0.85 (t, *J* = 6.8 Hz, 3H, H-6′), δ_H_ 1.68 (s br, 3H, H-29), and δ_H_ 1.21 (d, *J* = 6.30 Hz, 3H, H-28).

The ^13^C NMR spectrum contained 35 carbon signals; three methyl carbons sp^3^ at δ_C-28_ 20.29, δ_C-19_ 11.33, and δ_C-6′_ 14.38; 12 methylene groups sp^3^ at δ_C-4_ 42.47, δ_C-6_ 44.08, δ_C-8_ 44.32, δ_C-10_ 42.94, δ_C-12_ 44.94, δ_C-14_ 42.99, δ_C-26_ 37.98, δ_C-1′_ 29.47, δ_C-2′_ 28.98, δ_C-3′_ 27.13, δ_C-4′_31.55 and δ_C-5′_ 22.41; nine methine carbons sp^3^ at δ_C-2_ 53.21, δ_C-3_ 71.26, δ_C-5_ 69.98, δ_C-7_ 70.23, δ_C-9_ 71.53, δ_C-11_ 69.44, δ_C-13_ 65.67, δ_C-15_ 73.74, and δ_C-27_ 70.17; 10 carbon signals that were assigned to the olefin bond at δ_C-16_ 140.96, δ_C-17_ 126.28, δ_C-18_ 128.60, δ_C-19_ 133.22, δ_C-20_ 133.01, δ_C-21_ 133.27, δ_C-22_ 131.70, δ_C-23_ 133.39, δ_C-24_ 132.74, and δ_C-25_ 130.90. The quaternary carbon at 172.80 ppm was correlated with the lactone group C-1.

The structure of filipin I (Figure 1) was deduced by a combination of 1D and 2D NMR experiments. Analysis of COSY and HSQC–TOCSY (Figure 1) established the presence CH_3_-(CH_2_)_5_-CH-(CH-CH_2_)_6_-CH (C-6′to C-15) and (CH)_9_-CH_2_-CH-CH_3_ (C-17 to C-28) spin systems. In the HMBC spectrum (Figure 1), connections of C-1 with H-2 (δ_H_ 2.21), H-3, H-27 (δ_H_ 5.02), and H-1′ (δ_HA_ 1.68 and δ_HB_ 1.44) indicated the macrocyclic lactone structure. HMBC correlation of H-29 (δ_H_ 1.68) with C-15 (δ_C_ 73.74) and C-17 (δ_C_ 126.28) suggests that methyl group CH_3_-29 is connected with C-16. Other significant HMBC correlations for OH assignations of filipin I are given in Figure 1: OH-3 with C-2 (δ_C_ 53.21) and C-4 (δ_C_ 42.47), OH-5 with C-4 and C-6 (δ_C_ 44.08), OH-7 with C-6 and C-8 (δ_C_ 44.32), OH-9 with C-8 and C-10 (δ_C_ 42.94), OH-11 with C-10 and C-12 (δ_C_ 44.94), OH-13 with C-12 and C-14 (δ_C_ 42.94), and OH-15 with C-14 and C-16 (δ_C_ 140.96).

Detailed interpretation of COSY, HSQC, and HMBC experiments with filipin I (Figure 1, Table 1) have established that the hydroxyl groups are part of methane carbinols that form a highly oxygenated backbone structure consisting of a 1,3,5,7,9,11,13-heptahydroxy system (C-3, C-5, C-7, C-9, C-11, C-13, and C-15).

Filipin II (Figure 1) was revealed to have the molecular formula C_35_H_58_O_10_ by MS (*m/z* 661.5 [M+Na]^+^), ^13^C NMR, and ^1^H NMR (Figure S2). It exhibits a close similarity of carbon chemical shifts to those of filipin I (Table 1), with the exception of the carbon signal C-26 (δ_C_ 72.11) and the proton resonance at δ_H_ 3.93 (m) and δ_OH_ 5.26 (d, *J* = 5.6 Hz) corresponding to oxygenated methine proton H-26 from COSY and HSQC experiments. In addition, the HMBC correlations (Figure 1) from CH-26 to CH_3_-28 (δ_H_ 1.22, δ_C_ 18.54), as well as OH-26 (δ_H_ 5.26) with C-25 (δ_C_ 135.62) and C-27 (δ_C_ 73.40), clearly indicate the presence of a hydroxyl group at C-26.

Comparison of the spectrum of filipin I with that of filipin II (Table 1) indicates that the signal C-26/H-26 retained the hydroxyl function in the spectrum of filipin II (δ_H_ 3.93, δ_OH_ 5.26, and δ_C_ 72.11), while the hydroxyl function was absent in the spectrum of filipin I: δ_Ha_ 2.35, δ_Hb_ 2.25, and δ_C_ 37.98.

Stereochemical assignment of filipin I and filipin II has been established here by comparison with NMR data from filipin III. A conformational study of filipin III based on circular dichroism spectra [13] and its absolute stereochemistry, which was later and independently described based on chemical derivatizations [14,15], allowed the establishment of filipin III as 1′R, 2R, 3S, 5S, 7S, 9R, 11R, 13R, 15S, 26S, and 27R. Based on this assignment, the first total stereoselective synthesis was published [16]. Filipin III was reinvestigated based on NMR data, in order to assign ^1^H and ^13^C NMR signals [17,18]. In this context, we report the first-time complete assignments of ^1^H and ^13^C NMR of filipin I and filipin II.

### 2.2. Antifungal Activity

**Figure 2:**
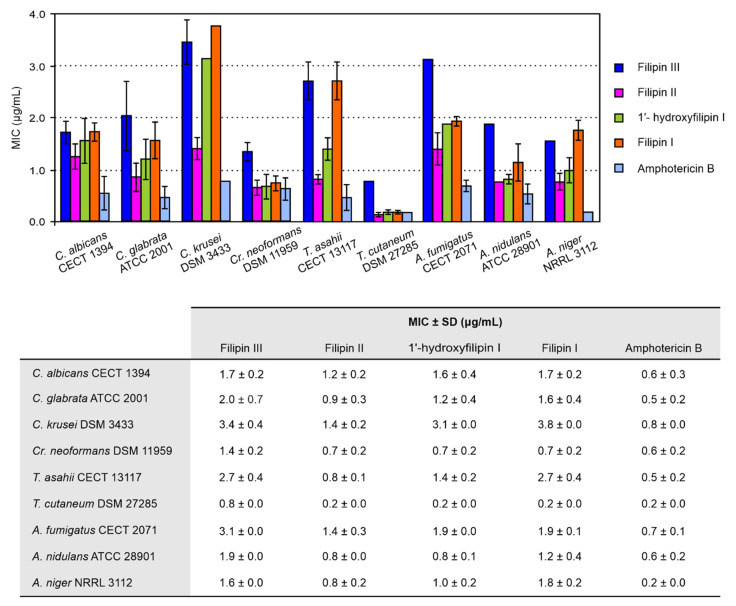
Minimum inhibitory concentrations (MICs) of filipin III intermediates. Data are the average of three determinations from independent experiments. MICs of amphotericin B were determined under identical conditions. Vertical bars indicate the standard deviation (SD) values. All compounds were fungicidal at the MIC.

The lower minimum inhibitory concentrations (MICs) of both filipin II and 1′-hydroxyfilipin I, when compared with that of filipin III for *C. utilis* and *S. cerevisiae* reported previously [12], prompted us to investigate the MICs of the different intermediates against a battery of yeasts and filamentous fungi known for being opportunistic pathogens. Six yeasts and three filamentous fungi were chosen for the analyses, including *C. albicans*, *C. glabrata*, *C. krusei*, *Cr. neoformans*, *T. cutaneum*, *T. asahii*, *A. nidulans*, *A. niger*, and *A. fumigatus*. To remove impurities from the polyene samples used for structural characterization, polyene intermediates were further purified by HPLC, as indicated in Materials and Methods. Figure S3 shows the chromatograms of the different intermediates used in the assays and results, which are summarized in Figure 2.

Our results agreed with our previous observations [12], being that filipin I and filipin III are the least active against most strains. Filipin II turned out to yield the lowest MICs of all the intermediates for all the strains tested, followed by 1′-hydroxyfilipin I, but with higher values than amphotericin B, the reference standard. In any case, all the intermediates showed potent antifungal activity. All intermediates were fungicidal for each strain at the MIC. Noteworthily, analysis of the antifungal activity of 1′-hydroxyfilipin I and filipin I revealed a MIC of 0.7 μg/mL against *Cr. neoformans*, which indicates a potency very similar to that of the current most effective antifungal agent against cryptococcosis amphotericin B (0.6 μg/mL under the same conditions), while displaying substantially lower haemolytic activity (see below).

### 2.3. Haemolytic Activity

**Figure 3:**
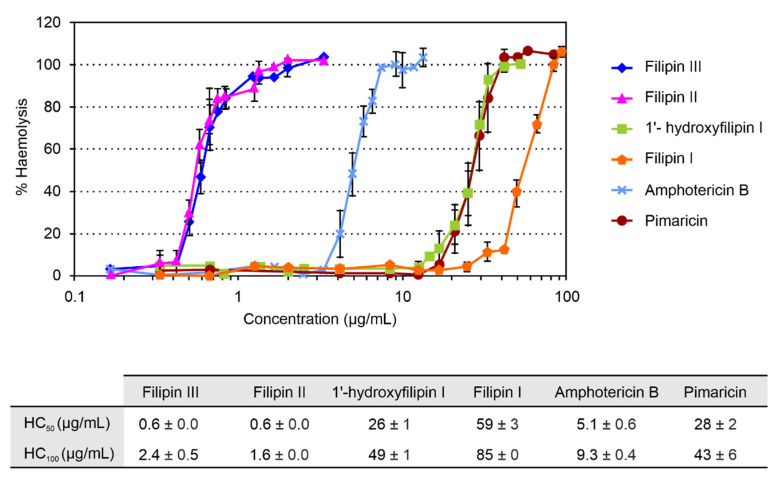
Haemolytic activity of filipin III and its intermediates. Haemolytic activity of pimaricin and amphotericin B was determined under identical conditions for comparison. Haemolytic activity caused by pure water was considered as 100%. Data are the average of three determinations from independent experiments. Vertical bars indicate the standard deviation values.

Given that the main disadvantage of polyene macrolides is their host toxicity, because of their haemolytic action on mammalian cells, we studied the haemolytic activity of the different intermediates by determining their haemolytic concentrations (HCs) and comparing them with those of the antifungal reference standard amphotericin and the tetraene pimaricin, a potent antifungal polyene with a GRAS (Generally Recognized As Safe) status widely used as a food preservative [19] (Figure 3). Filipin II showed similar levels of toxicity to filipin III, causing total haemolysis at concentrations below 2 μg/mL. However, 1′-hydroxyfilipin I was found to be much less toxic, requiring concentrations 20 times higher to reach 100% haemolysis (HC_50_ 26 μg/mL; HC_100_ 49 μg/mL). In the case of filipin I, the first precursor in filipin III biosynthesis, its HC_50_ was 59 μg/mL. These two latter compounds showed substantially reduced haemolytic values when compared to amphotericin B, around five-fold lower in the case of 1′-hydroxyfilipin I, and more than nine-fold in the case of filipin I (Figure 3). It is worth noting that the haemolytic activity of 1′-hydroxyfilipin I was comparable to that of pimaricin, and that of filipin I even lower, displaying values about half of that provoked by the tetraene (Figure 3). 

The only structural difference between the four filipins studied is the presence or absence of hydroxyl groups at positions C26 and C1′ (Figure 1). Thus, the most haemolytic filipins (filipin II and filipin III) present a hydroxyl group at C26 of the macrocyclic ring that is absent in both 1′-hydroxyfilipin I and filipin I. This suggests that the C26 hydroxyl group of the filipins is a main determinant for their affinity for cholesterol. Toxicity reduction by deletion of a single hydroxyl group has been reported with other compounds. Substitution of a hydroxyl group by a ketone group in bile salts produces a significant decrease in their membranolytic activity [20]. Haemolytic activity of polyene amphotericin B is drastically reduced when the hydroxyl group at C2′ of mycosamine is eliminated [21]. At high concentrations, the toxicity could be caused by a more unspecific effect of the hydroxyl group at the C1′ position, which is reflected in the 40-fold lower toxicity (HC_50_) of 1′-hydroxyfilipin compared to filipin II or filipin III. Even higher concentrations of filipin I, which lacks a C1′ hydroxyl, are needed to observe haemolytic activity (Figure 3), probably because filipin I lacks both hydroxyl groups. However, the striking thing about 1′-hydroxyfilipin I and filipin I is that they show reduced toxicity while retaining antifungal activity. Other polyene macrolides, such as some nystatin derivatives (e.g., BSG003 and BSG018), show dramatically reduced haemolytic activities upon hydroxyl group modifications; in this case, though, antifungal activity is also substantially reduced [22].

## 3. Discussion

Fungal infections constitute a worldwide problem, in particular in the expanding population of immunocompromised individuals in which such infections often become invasive, with devastating consequences [23]. The main agents responsible for these infections are members of the genera *Candida*, *Cryptococcus*, and *Aspergillus*, although infections caused by other fungi are becoming more frequent every year [24].

Polyene macrolides are broad-spectrum antifungal agents with strong fungicidal activity. Their molecular target is ergosterol, the main sterol of fungal membranes, and their action is thought to be derived from the alteration of the normal functioning of ergosterol in the membrane [19,25]. Because ergosterol is an essential structural constituent of the membrane, polyenes are extremely reluctant to provoke microbial resistance [26]. Their main limitation, however, is host toxicity—particularly towards kidney cells that are especially sensitive to the haemolytic action of polyenes, which is due to the similarity between ergosterol and cholesterol in the mammalian cell membrane [27]. Luckily, the introduction of lipid-complexed formulations of polyenes has managed to considerably reduce this toxicity [28].

Among polyenes, filipin III is particularly toxic, because it shows a similar affinity for ergosterol and cholesterol [11]. Here we have provided evidence that the haemolytic action of filipin III (and that of its derivatives) relies largely on the presence of a hydroxyl function at C26, since derivatives lacking a hydroxyl function at such a position, such as filipin I and 1′-hydroxyfilipin I, show remarkably reduced haemolytic action on horse erythrocytes. Given that these latter intermediates retain potent antifungal activity, their affinity for cholesterol must be considerably reduced. In fact, the haemolytic activity of 1′-hydroxyfilipin I on horse erythrocytes is similar to that of pimaricin, a glycosylated tetraene with a strong preference for ergosterol over cholesterol [11]. Taking into consideration that 1′-hydroxyfilipin I has a reduced MIC on the majority of fungal strains tested when compared to filipin I, it makes this derivative a promising lead for the development of efficient pharmaceuticals. The antifungal potency of 1′-hydroxyfilipin I is lower than that of amphotericin B on most strains, but its haemolytic activity is five times lower.

Particularly attractive is the effect of 1′-hydroxyfilipin I and filipin I on *Cr. neoformans*. Cryptococcosis is a neglected fungal meningitis that causes approximately half a million deaths annually [29]. The most effective antifungal agent against the disease is amphotericin B, but it is highly toxic. The effectiveness of this drug is thought to rely on its fungicidal activity rather than just halting growth. Filipin I and 1′-hydroxyfilipin I are fungicidal polyenes, like amphotericin B, and have similar MICs for *Cr. neoformans*; however, their toxicity is between five and nine times lower than amphotericin B, and thus they could constitute excellent alternatives for the successful treatment of this form of meningitis.

Normally, the final product of a biosynthetic pathway is the compound with highest bioactivity [30,31], but this is not the case with filipin III intermediates. We previously proposed that this could be due to an improved affinity for ergosterol of such intermediates when compared to filipin III [12]; we have now demonstrated that this is in fact the case for 1′-hydroxyfilipin I and filipin I on some fungal strains, but our results also indicate that these compounds show reduced affinity for cholesterol, resulting in a drastic reduction of haemolytic values compared to filipin III.

## 4. Materials and Methods 

### 4.1. Microbial Strains and Cultivation

Recombinant *S. filipinensis ΔfilC*, *ΔfilD*, and *ΔfilCD* strains [12] were used as a source of filipin III derivatives 1′-hydroxyfilipin I, filipin II, and filipin I, respectively. *Streptomyces* strains were routinely grown in yeast extract–malt extract (YEME) medium [32] without sucrose. The sporulation of *Streptomyces* strains was achieved as described elsewhere [33]. *Candida albicans* CECT 1394, *C. glabrata* ATCC 2001, *C. krusei* (syn. *Issatchenkia orientalis*) DSM 3433, *Cryptococcus neoformans* DSM 11959, *Trichosporon cutaneum* DSM 27285, *T. asahii* CECT 13117, *Aspergillus awamori* (syn. *A. niger* var. fusca) NRRL 3112, *A. fumigatus* CECT 2071, and *A. nidulans* ATCC 28901 were used for susceptibility experiments. Yeast strains were routinely grown in Sabouraud dextrose agar medium (SDA; ADSA Micro), whereas for filamentous fungi we used potato dextrose agar (PDA; Difco). 

### 4.2. Filipin Derivative Purification 

For the structural determination of filipin derivatives, *S. filipinensis* strains were grown over 72 h at 250 rpm and 30 °C. Fermentation broths were then harvested by centrifugation, and the supernatant extracted with two volumes of ethyl acetate. The organic phase was then sequentially treated with saturated NaCl solution and Na_2_SO_4_, concentrated by rotaevaporation, vacuum-dried, and resuspended in pure methanol. Filipin derivatives were further purified by reverse phase HPLC, as described elsewhere [12], for bioactivity determinations. Peaks with the appropriate retention times were collected and pooled. The retention times for filipin III, filipin II, 1′-hydroxyfilipin I, and filipin I were 12.5, 14.5, 15.7, and 17.3 min, respectively (Figure S3). 

### 4.3. Structural Elucidation of Compounds

The structure elucidation of filipin I and filipin II was carried out by MS, followed by 1D and 2D NMR spectroscopy. For MS analyses, the purified filipin derivative was dissolved in methanol, whereas for NMR determination it was dissolved in deuterated dimethyl sulfoxide. 

Mass spectra were obtained on an Ultrafex III MALDI TOF/TOF apparatus (Bruker), using ditranol + NaI as the matrix, and the FAB-MS spectrum was recorded on a VG Autospectrum (Waters) instrument using *m*-nitrobenzyl alcohol (*m*-NBA) as the matrix and caesium (Cs+) as the ion bombardment at 35 KV. A filipin III solution (5 mg/mL methanol) was used for tuning. 

Structures were elucidated on the basis of the 1D NMR, including ^1^H-NMR, ^13^C-NMR, and DEPT (distortionless enhancement by polarization transfer), as well as 2D-NMR, including COSY (H,H-correlation spectroscopy), HSQC (heteronuclear single quantum coherence), HMQC (heteronuclear multiple quantum correlation), and HMBC (heteronuclear multiple-bond correlation) experiments; they were also determined by comparison with the spectrum of filipin III [17]. NMR spectra were recorded in DMSO-*d*6 at room temperature using a Bruker WM 500 spectrometer (500 MHz (^1^H-NMR) and 125 MHz (^13^C-NMR)). The pulse programmes of the two-dimensional experiments were taken from the Bruker software library, and the parameters were as follows. The 500/125 MHz gradient-selected HMQC spectra: relaxation delay *D*1 = 1.5 s; the 500/125 MHz gradient-selected HMBC spectra: relaxation delay *D*1 = 1.5 s, evolution delay *D*2 = 3.33 ms, delay for evolution of long-range coupling *D*6 = 60 ms; the 500 MHz gradient-selected ^1^H,^1^H COSY spectra: relaxation delay *D*1 = 1.5 s; 90° pulse for ^1^H.

### 4.4. MIC and Minimal Fungicide Concentration (MFC) Determination

Minimum inhibitory concentrations (MICs) were determined by the broth microdilution technique, following the Clinical and Laboratory Standards Institute (CLSI) guidelines by diluting freshly prepared filipin derivatives in Roswell Park Memorial Institute (RPMI) 1640 medium with glutamine and 0.2% glucose, but without sodium bicarbonate (Sigma), buffered with 0.164 M MOPS pH 7.0 to concentrations of 60, 50, or 40 μg/mL, of which 100 μL was added to the first row of a round-bottomed, 96-well culture plate. This was followed by a 1:1 dilution series in medium. Yeast antifungal susceptibility testing was performed according to the M27-A3 protocol [34], whereas for filamentous fungi testing we used the M38-A2 method [35]. The MIC value was determined to be the lowest concentration of antibiotic that inhibited completely the growth of the fungi and could be determined by the eye on the 96-well plate after an incubation of 48–72 h at 35 °C. Commercial filipin III (Sigma) and amphotericin B (Sigma) were used as controls. 

For minimal fungicidal concentration (MFC) determination, the total volume of each well used for MIC determination, starting from the last well in which growth was observed up to the highest drug concentration tested, was transferred onto agar plates. The MFC corresponded to the lowest drug concentration at which no colonies were observed after 48–72 h of incubation.

MIC and MFC values were determined as the average of three determinations from independent experiments. The MFC/MIC ratio was calculated to determine whether the substances exhibited fungistatic (MFC/MIC > 4) or fungicidal activity (MFC/MIC < 4) [36].

### 4.5. Haemolytic Activity Assay 

Haemolytic activity was determined as described elsewhere [37]. Briefly, the polyene samples were weighed and dissolved in pure dimethyl sulfoxide (DMSO) at 1 mg/mL. Increasing quantities of the different polyenes (between 0 and 600 µg/mL) were brought to a final volume of 100 μL of DMSO and mixed gently with 500 μL of phosphate-buffered saline (PBS) containing 2.5% defibrinated horse blood (Oxoid). After incubation at 37 °C for 30 min without agitation, cells were pelleted by centrifugation, and haemolysis evaluated by measuring the absorbance at 545 nm in a Hitachi U-2000 spectrophotometer. Commercial antifungal polyenes filipin III (Sigma), amphotericin B (Sigma), and pimaricin (Sigma) were used for comparison. The values corresponding to total haemolysis were estimated with a suspension of 2.5% horse blood in distilled water. A sample with 100 μL DMSO was used as a control.

## Figures and Tables

**Table 1:** ^1^H and ^13^C NMR chemical shifts assignments for filipin I and filipin II (DMSO-d_6_).

C	Filipin I		Filipin II	
	δ_C_ (DEPT)	δ_H_	δ_C_ (DEPT)	δ_H_
1	172.80 (C)		173.70 (C)	
2	53.21 (CH)	2.21 (m)	53.00 (CH)	2.25 (m)
3	71.26 (CH)	3.61 (m); 5.05 (d, *J* = 4.8 Hz, OH)	71.32 (CH)	3.63 (m); 5.02 (d, *J* = 4.4 Hz, OH)
4	42.47 (CH_2_)	1.30 (m)	42.17 (CH_2_)	1.32 (m)
5	69.98 (CH)	3.83 (m); 4.97 (d, *J* = 2.2 Hz OH)	70.01 (CH)	3.88 (m); 5.00 (d, *J* = 2.0 Hz, OH)
6	44.08 (CH_2_)	1.29 (m)	44.08 (CH_2_)	1.30 (m)
7	70.23 (CH)	3.85 (m); 4.89 (d, *J* = 1.8 Hz, OH)	70.33 (CH)	3.88 (m); 4.95 (d, *J* = 1.8 Hz, OH)
8	44.32 (CH_2_)	A: 1.38 (m) B: 1.28 (m)	44.32 (CH_2_)	A: 1.40 (m) B: 1.30 (m)
9	71.53 (CH)	3.82 (m); 5.13 (s br, OH)	71.49 (CH)	3.85 (m); 5.15 (d, *J* = 1.4 Hz, OH)
10	42.94 (CH_2_)	A: 1.35 (m) B: 1.22 (m)	43.05 (CH_2_)	A: 1.36 (m) B: 1.28 (m)
11	69.44 (CH)	3.79 (m); 4.84 (d, *J* = 2.2 Hz, OH)	69.45 (CH)	3.83 (m); 4.83 (d, *J* = 2.5 Hz, OH)
12	44.94 (CH_2_)	A: 1.59 (m) B: 1.28 (m)	44.86 (CH_2_)	A: 1.61 (m) B: 1.28 (m)
13	65.67 (CH)	3.18 (m); 4.46 (d, *J* = 4.8 Hz, OH)	65.76 (CH)	3.09 (m); 4.43 (d, *J* = 4.7 Hz, OH)
14	42.99 (CH_2_)	A: 1.71 (m) B: 1.52 (m)	43.19 (CH_2_)	A: 1.71 (m) B: 1.53 (m)
15	73.74 (CH)	3.98 (m); 4.82 (d, *J* = 3.7 Hz, OH)	73.77 (CH)	3.98 (m); 4.79 (d, *J* = 3.7 Hz, OH)
16	140.96 (C)		141.09 (C)	
17	126.28 (CH)	5.93 (d, *J* = 11.2 Hz)	126.10 (CH)	5.93 (d, *J* = 11.2 Hz)
18	128.60 (CH)	6.46 (dd, *J* = 14.5, 11.2 Hz)	128.71 (CH)	6.48 (dd, *J* = 14.5, 11.2 Hz)
19	133.22 (CH)	6.23 (m)	133.18 (CH)	6.22 (m)
20	133.01 (CH)	6.35 (m)	133.08 (CH)	6.33–6.36 (m)
21	133.27 (CH)	6.31 (m)	132.26 (CH)	6.27 (m)
22	131.70 (CH)	6.18 (m)	133.15 (CH)	6.33–6.36 (m)
23	133.39 (CH)	6.28 (m)	133.37 (CH)	6.33–6.36 (m)
24	132.74 (CH)	6.13 (dd, *J* = 14.5, 11.2 Hz)	129.86 (CH)	6.31 (m)
25	130.90 (CH)	5.75 (dt, *J* = 14.5, 6.9 Hz)	135.62 (CH)	5.92 (d, *J* = 14.5 Hz)
26	37.98 (CH_2_)	A: 2.35 (m) B: 2.25 (m)	72.11 (CH)	3.93 (m); 5.26 (d, *J* = 5.6 Hz, OH)
27	70.17 (CH)	5.02 (m)	73.40 (CH)	4.63 (m)
28	20.29 (CH_3_)	1.21 (d, *J* = 6.30 Hz)	18.54 (CH_3_)	1.22 (d, *J* = 6.20 Hz)
29	11.33 (CH_3_)	1.68 (s br)	11.46 (CH_3_)	1.69 (s br)
1′	29.47 (CH_2_)	A: 1.68 (m) B: 1.44 (m)	29.60 (CH_2_)	A: 1.68 (m) B: 1.43 (m)
2′	28.98 (CH_2_)	1.24 (m)	29.04 (CH_2_)	1.24 (m)
3′	27.13 (CH_2_)	1.19 (m)	27.05 (CH_2_)	1.19 (m)
4′	31.55 (CH_2_)	1.22 (m)	31.59 (CH_2_)	1.22 (m)
5′	22.41 (CH_2_)	1.24 (m)	22.45 (CH_2_)	1.25 (m)
6′	14.38 (CH_3_)	0.85 (t, *J* = 6.8 Hz)	14.40 (CH_3_)	0.86 (t, *J* = 6.8 Hz)

## Supplementary Materials

The following are available online at https://www.mdpi.com/2079-6382/9/7/413/s1, Figure S1: Filipin I spectra: (A) MS MALDI, (B) ^1^H NMR, (C) ^13^C NMR, (D) HSQC, (E) HMBC, and (F) COSY. Figure S2: Filipin II spectra: (A) MS MALDI, (B) ^1^H NMR, (C) ^13^C NMR, (D) HMBC, (E) HSQC, and (F) COSY. Figure S3: HPLC chromatograms of purified derivatives used in bioactivity assays.
