# Supplementary material for: Structural and Bioactivity Characterization of Filipin Derivatives from Engineered Streptomyces filipinensis Strains Reveals Clues for Reduced Haemolytic Action
Source: Antibiotics (Basel). 2020 Jul 16;9(7):413. doi: 10.3390/antibiotics9070413 (PMC7400637; doi:10.3390/antibiotics9070413)
Supplement: Supplementary file 1 [file antibiotics-09-00413-s001.pdf]

**Supplementary information**

# Structural and bioactivity characterization of filipin derivatives from engineered *Streptomyces filipinensis* strains reveals clues for reduced haemolytic action.

**Eva G. Barreales [1], Ángel Rumbero [2], Tamara D. Payero [1], Antonio de Pedro [1], Ester Jambrina [1] and Jesús F. Aparicio [1,*]**

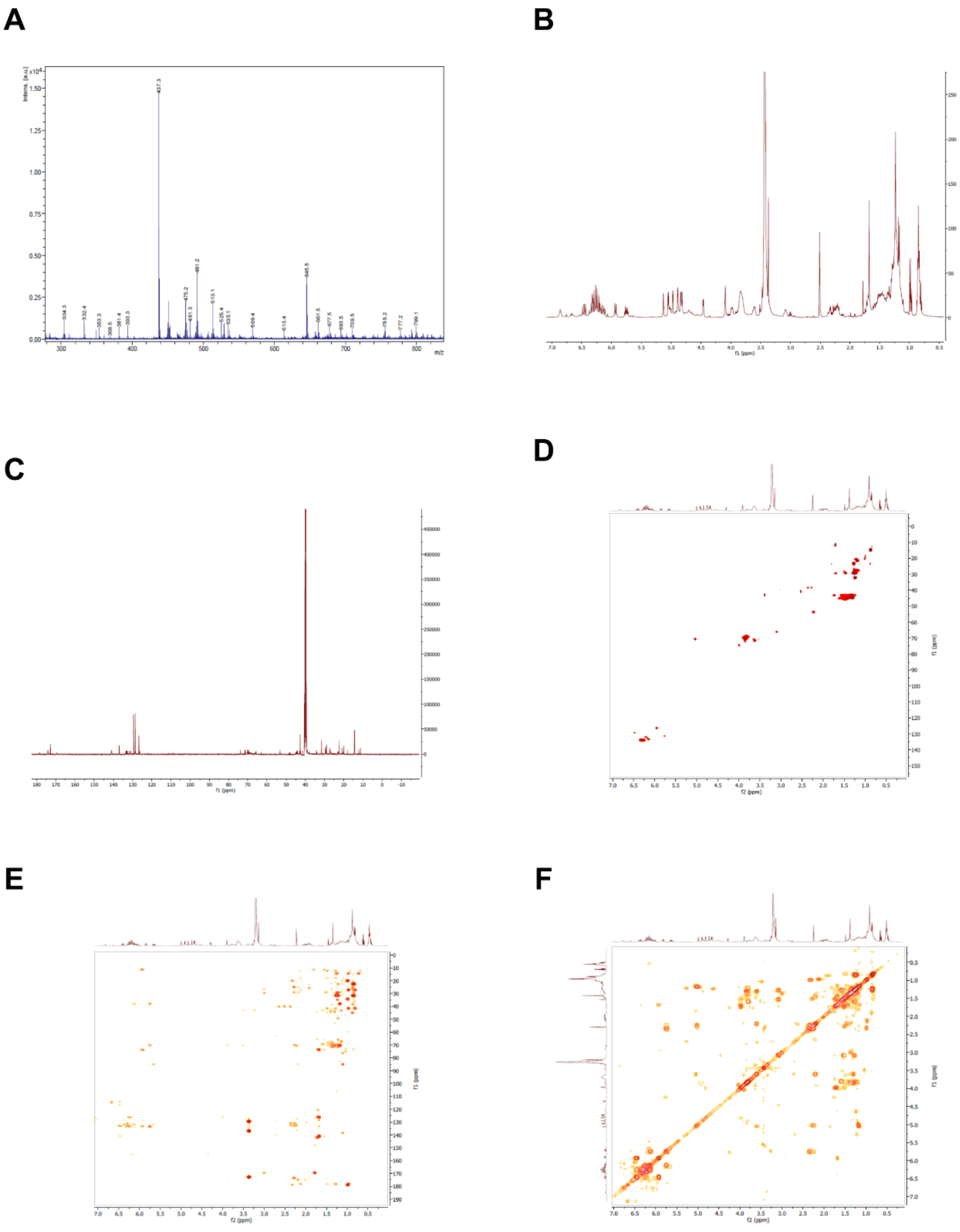


Figure S1: Filipin I spectra: A) MS MALDI, B) 1H NMR, C) 13C NMR, D) HSQC, E) HMBC, F) COSY.

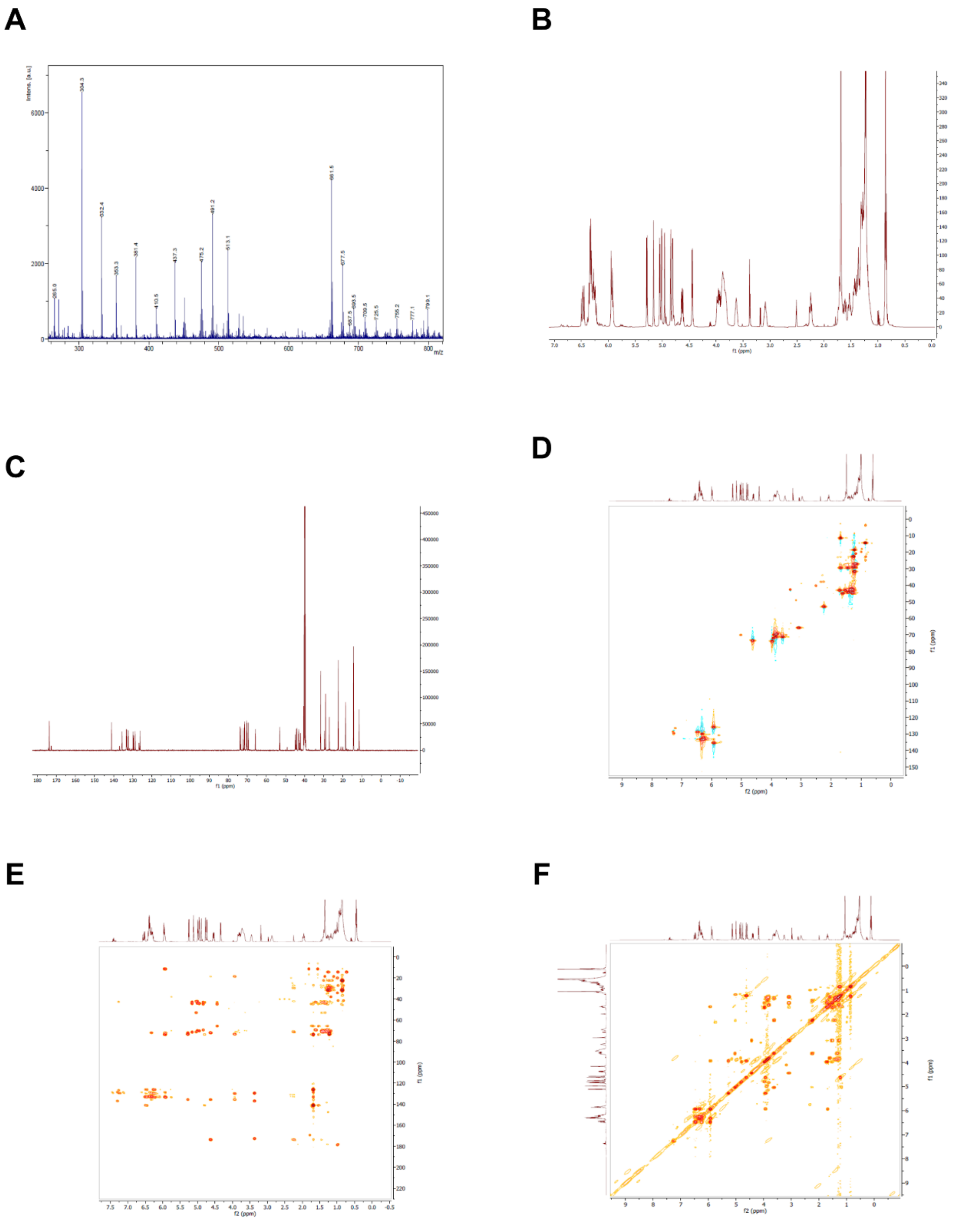


Figure S2: Filipin II spectra: A) MS MALDI, B) 1H NMR, C) 13C NMR, D) HMBC, E) HSQC, F) COSY.

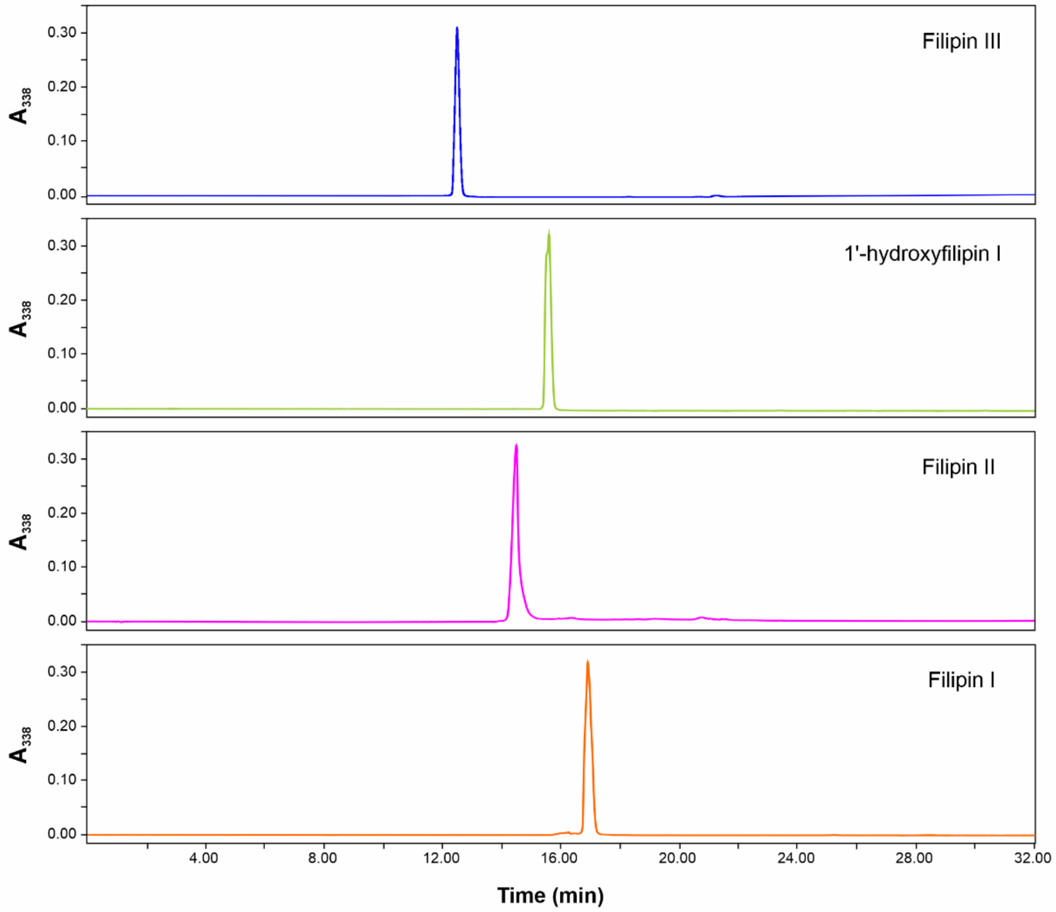


Figure S3: HPLC chromatograms of purified derivatives used in bioactivity assays.
